# The effect of ozone fumigation on the biogenic volatile organic compounds (BVOCs) emitted from *Brassica napus* above- and below-ground

**DOI:** 10.1371/journal.pone.0208825

**Published:** 2018-12-10

**Authors:** W. J. F. Acton, W. Jud, A. Ghirardo, G. Wohlfahrt, C. N. Hewitt, J. E. Taylor, A. Hansel

**Affiliations:** 1 Lancaster Environment Centre, Lancaster University, Lancaster, United Kingdom; 2 Institute of Ion and Applied Physics, University of Innsbruck, Innsbruck, Austria; 3 Helmholtz Zentrum München, Institute of Biochemical Plant Pathology, Research Unit Environmental Simulation (EUS), Neuherberg, Germany; 4 Institute of Ecology, University of Innsbruck, Innsbruck, Austria; University of Copenhagen, DENMARK

## Abstract

The emissions of BVOCs from oilseed rape (*Brassica napus*), both when the plant is exposed to clean air and when it is fumigated with ozone at environmentally-relevant mixing ratios (ca. 135 ppbv), were measured under controlled laboratory conditions. Emissions of BVOCs were recorded from combined leaf and root chambers using a recently developed Selective Reagent Ionisation—Time of Flight—Mass Spectrometer (SRI-ToF-MS) enabling BVOC detection with high time and mass resolution, together with the ability to identify certain molecular functionality. Emissions of BVOCs from below-ground were found to be dominated by sulfur compounds including methanethiol, dimethyl disulfide and dimethyl sulfide, and these emissions did not change following fumigation of the plant with ozone. Emissions from above-ground plant organs exposed to clean air were dominated by methanol, monoterpenes, 4-oxopentanal and methanethiol. Ozone fumigation of the plants caused a rapid decrease in monoterpene and sesquiterpene concentrations in the leaf chamber and increased concentrations of ca. 20 oxygenated species, almost doubling the total carbon lost by the plant leaves as volatiles. The drop in sesquiterpenes concentrations was attributed to ozonolysis occurring to a major extent on the leaf surface. The drop in monoterpene concentrations was attributed to gas phase reactions with OH radicals deriving from ozonolysis reactions. As plant-emitted terpenoids have been shown to play a role in plant-plant and plant-insect signalling, the rapid loss of these species in the air surrounding the plants during photochemical pollution episodes may have a significant impact on plant-plant and plant-insect communications.

## Introduction

Biogenic volatile organic compounds (BVOCs) are a large and diverse group of molecules released from plants into the atmosphere [1]. Plants have been shown to emit BVOC from both above- and below-ground organs and these emissions are known to change in response to both biotic and abiotic stress [2]. These BVOCs represent a major source of reactive carbon released into the atmosphere, with ca. 10^15^ g emitted annually [3], and hence they play a significant role in tropospheric chemistry both by acting as a sink for atmospheric oxidants, such as OH radicals, and through their effects on the formation of secondary organic aerosol (SOA) and tropospheric ozone [4]. BVOCs, therefore, significantly impact both the climate system and local-to-regional scale air quality.

Ozone is formed photochemically in the troposphere by reactions involving volatile organic compounds (VOCs) and NO_x_ (NO and NO_2_) [4–5]. In the northern hemisphere background tropospheric ozone levels are generally in the range 35–40 ppbv, but locally can peak above 100 ppbv depending on concentrations of precursors and weather conditions [6]. Recent studies indicate that peak tropospheric ozone concentrations have stabilised, or are decreasing, in some industrialised areas due to reductions in precursor emissions [7]. However, ozone concentrations in the troposphere continue to rise in East Asia [8]. Tropospheric ozone is an especially important pollutant due to the detrimental impact it has been shown to have on both human health and crop yields [6, 9–10].

BVOC emissions from plants are affected by both biotic stress and the environmental conditions to which the plant is exposed [2, 11], including the ambient concentrations of ozone. BVOCs emitted by plants have been shown to protect plants from harmful ozone-induced oxidative stress [12–13]. The impact of ozone on the emission of BVOCs from plants has been shown to be dependent on the sensitivity of the plant species to ozone [14–15], the plant/leaf growth stage [16], the specific BVOCs emitted [17–19], the concentration of ozone and whether ozone exposure is acute or chronic [14, 20].

A change in both the quantity of BVOCs emitted and the chemical composition of the BVOC blend, is also commonly observed in response to biotic stresses [21]. Both constitutive and induced BVOC emissions can act as a direct defence, repelling herbivores, or as an indirect defence through a “cry for help” [22–23] to attract the predators of the herbivorous organisms attacking them. BVOC emitted during or after the occurrence of biotic stress include a large variety of different mono- and sesqui-terpenes [24–25], which have been shown to play important roles in plant signalling above [23,26–27] and below ground [28]. There have been limited studies on the effect of ozone on plant BVOC signalling but the few available studies suggest that ozone may affect plant-insect signalling [29–30]. Upon exposure to ozone, BVOCs containing an alkene functional group, such as monoterpenes and sesquiterpenes, may undergo ozonolysis in the gas, liquid, or condensed phase, respectively, inside or outside of the plant [31]. The gas phase ozonolysis of alkenes leads to the formation of an energy-rich ozonide, which rapidly decomposes to a carbonyl and a high energy Criegee intermediate [32]. The Criegee intermediate can be collisionally-stabilised or, depending on the structure of the Criegee intermediate, can decay to either CO_2_ + RH or to an OH radical and a substituted alkyl radical [33]. Thus the ozonolysis of terpenoids can act as a source of OH and a range of oxygenated BVOCs.

Here we aim to test the impact of above ground ozone fumigation on BVOC emission from *Brassica napus* (oilseed rape) both above and below ground parts. We report, for the first time, the BVOCs emitted from the whole plant measured simultaneously above and below ground, using the recently developed selective reagent ionisation-time of flight-mass spectrometer (SRI-ToF-MS). The effect of ozone fumigation on BVOC emissions both above- and below-ground is reported. Using the highly time-resolved and sensitive SRI-ToF-MS, we could follow the initial reaction of several biogenically emitted terpenoids with ozone and observed the formation of a number of atmospherically-abundant oxygenated compounds. *Brassica napus* (oilseed rape) was selected as the model plant species due to its wide geographic distribution, its importance as a crop [34] and its known emission of both monoterpenes and sesquiterpenes [35].

## Materials and methods

Experiments were carried out in two blocks, July-September 2013 and April-June 2014. In 2013, BVOC emissions from potted plants enclosed in chambers were recorded from both plant leaves and from below ground using a SRI-ToF-MS. During the 2014 experiments, measurements were made from above ground alone using a SRI-ToF-MS in order to increase the time resolution of the leaf emission data. Additionally, BVOC samples were collected from the leaf chamber for chemical identification by GC-MS analysis.

### Plant material

*Brassica napus* plants (DK Cabernet, Monsanto) were grown from seed under natural light with supplementary heating in glass houses at the University of Innsbruck Botanical Gardens from May-July 2013 and January-April 2014. The average temperature within the glass houses was 19°C, the average relative humidity was 73% and the photoperiod was ca. 15.5 h in May-July and ca. 11 h in January-April. The soil used was made up of steamed leaf mould (31%), steamed basic soil (15%), coconut fibre (15%), sand (15%), rock flour (15%) and lava (12%). Measurements were carried out when plants were at growth stages 1.4–1.6 [36] meaning that plants had between 3 and 5 open adult leaves and had not yet undergone stem extension. Plants were transplanted prior to experiments into 2 L root chambers using the same soil and given a week to acclimatise to lab conditions. In 2013 four replicates were carried out using unique plants and in 2014 a further six replicates were carried out using unique plants.

### Experimental setup

**Fig 1:**
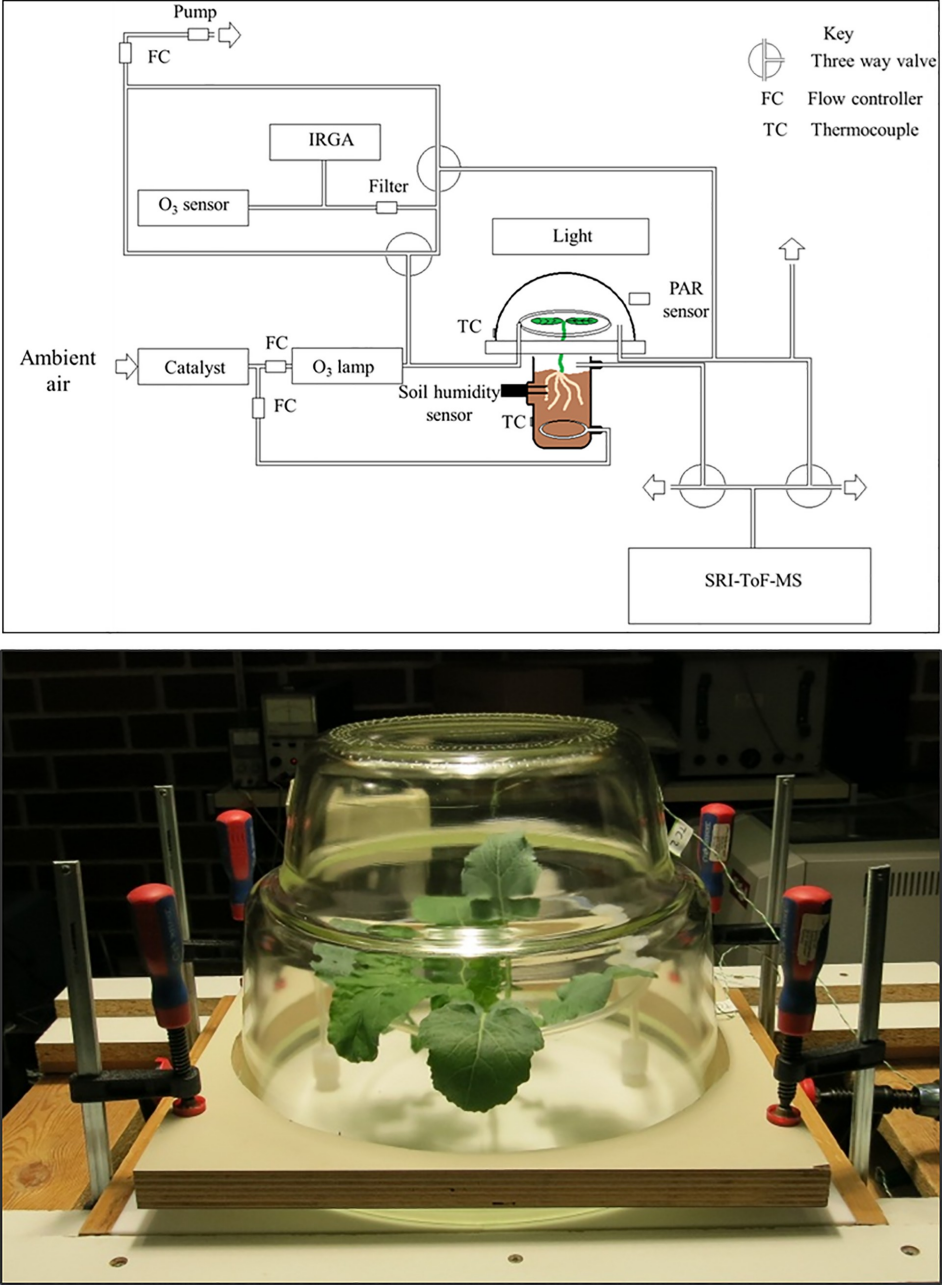
Schematic diagram of experimental setup (top) and plant chamber (bottom).

The experimental setup was based on that described by [37] and is shown in Fig 1. Both the leaf and root chambers were designed with glass and PFA/PTFE surfaces and the sampling line from the chamber outlet to the SRI-ToF-MS was heated to 60°C to avoid water condensation and to decrease analyte adsorption onto the surface. Care was taken to avoid skin contact with the leaf and root chamber as well as the plant material in order to prevent artefact formation through the ozonolysis of lipids present on human skin [38–39]. All tubing was PFA or PEEK and air flow rates were controlled using mass flow controllers (Bronkhorst, Ruurlo, NL). VOC-free air was generated by passing ambient air through a catalyst (Zero Air Generator HPZA-7000, Parker Balston, Haverhill, USA) heated to 300°C. This air flow was then split between root and leaf chambers with ca. 0.5 L min^-1^ directed into the root chamber and ca. 3.0 L min^-1^ to the leaf chamber. Ozone was produced by passing the leaf chamber air stream through an ozone generator made up of a mercury lamp which photolyzed O_2_ to make ozone (UVP, Upland, CA, USA). Ozone fumigation of the leaf chamber began after a period of 1–3 h recording BVOC emission in clean (ozone free) air and continued for 2–5 h. In the case of the 2014 experiments ozone fumigation was repeated the following day for each plant.

The root chamber consisted of a 2 L glass vessel with 6.35 mm inlet ports 20 mm from the top and bottom of the vessel and a 32 mm port capped with a PTFE coated septum mid-way down the chamber. VOC-free air entered the root chamber through a ring of PFA tubing, pierced in many places, at the base of the chamber and exited the chamber through the top port situated above the soil level. Soil humidity was recorded via a soil water content sensor (SM300, Delta-T Devices Ltd., Cambridge, UK) inserted through the septum. The temperature was recorded via a thermocouple attached to the exterior of the chamber so as not to provide a reactive surface within the chamber.

The leaf chamber was made up of a 17.2 L glass chamber resting on two 400 × 200 × 15 mm PTFE plates with a semi-circle of radius 6.3 mm cut for the plant stem in each plate. As with the root chamber, VOC-free air was introduced to the leaf chamber through a ring of PFA tubing pierced in many places in order to facilitate air mixing. The PTFE plates and the glass leaf and root chambers were held in place by external clamps (see Fig 1). PTFE tape was used between the PTFE plates, around the plant stem, and between both the glass leaf and root chambers and the PTFE plates in order to prevent leaks. Following installation of every new plant into the chamber, >12 h before the start of measurements, tests were carried out to ensure that the air flow in the root chamber was independent of that in the leaf chamber and *vice versa*. An overflow PFA tube (ca. 3 m length, 6.35 mm diameter), was installed between the leaf chamber and the SRI-ToF-MS in order to prevent pressure changes when the SRI-ToF-MS was switched from leaf to root chamber measurements. Light was provided from a growth lamp (Dakar, MT / HQI-T/D, Lanzini Illuminazione, Brescia, Italy) positioned ca. 1 m above the chamber and a water bath was installed between the lamp and the growth chamber to filter infra-red radiation and so limit chamber heating. Light and temperature were monitored using a BF3 Sunshine Sensor (Delta-T Devices Ltd., Cambridge, UK) and a thermocouple beside and affixed to the exterior of the leaf chamber respectively. The temperature difference between the inside and exterior of the leaf chamber was tested and found to be ca. 2°C when the growth lamp was on.

Approximately 1 L min^-1^ of air was subsampled before and after passing through the leaf chamber for analysis of O_3_ (ozone analyser, Model 49i, Thermo Fisher Scientific Inc. Franklin (MA), USA), CO_2_ and H_2_O (LI-840A CO_2_/H_2_O Analyzer, LI-COR Inc., Lincoln (NE), USA) concentrations. Measurements of these gases were switched between the inlet and outlet every two minutes. In order to maintain a constant rate of flow into the chamber and to the SRI-ToF-MS, when air was subsampled from the chamber outlet the same volume was pumped from the inlet line and *vice versa* (see Fig 1). The environmental conditions within the leaf chamber during both sets of experiments are summarized in Table 1.

**Table 1:** A summary of leaf chamber conditions and soil water content in root chamber. Values in parentheses represent the standard error between repeats. Ozone mixing ratios are the mean of those measured at the inlet and outlet of the leaf chamber.

	Average soil water content (%)	Temperature (°C)	PAR (μmol m^-2^ s^-1^)	CO_2_ (ppm)	H_2_O (parts per thousand)	O_3_ background (ppb)	O_3_ fumigation (ppb)
Summer 2013	43.7 (4.2)	26.6 (0.1)	424.3 (4.6)	333.1 (5.95)	19.9 (0.68)	2.5 (0.66)	138.8 (2.6)
Spring 2014	Root chamber not in use	26.9 (0.9)	400.0 (4.7)	345.8 (4.7)	16.2 (0.93)	0.0 (0.0)	143.8 (7.0)

In order to observe the effect of OH radical formation in the leaf chamber on BVOC emission an excess of cyclohexane was introduced into the leaf chamber. Cyclohexane is known to act as an efficient scavenger of OH, reacting with OH to give cyclohexanone and cyclohexanol [40]. As cyclohexane has a proton affinity less than that of water an excess may be introduced without disrupting the BVOC measurements made using SRI-TOF-MS in H_3_O^+^ mode. An excess of cyclohexane (Sigma-Aldrich, Steinheim, Germany) was introduced using a syringe via a septum into the gas inlet line immediately before the chamber in three of the spring 2014 experiments. This provided a burst of cyclohexane, removing OH radicals from the leaf chamber for approximately 20 mins (determined by monitoring the formation of cyclohexanone). Cyclohexanone has a proton affinity of 841 kJ mol^-1^, greater than that of water, so can be monitored using SRI-TOF-MS.

Background measurements were made weekly with the leaf chamber background consisting of the empty leaf chamber with and without ozone. Soil chamber backgrounds were made using two soil chambers filled with soil then watered and cared for in the same manner as those containing plants.

### SRI-ToF-MS measurements

BVOC measurements were made using the University of Innsbruck SRI-ToF-MS. This instrument is based on the proton transfer reaction time-of-flight mass spectrometer (PTR-ToF-MS) developed at the University of Innsbruck [41], with the additional ability to ionise analyte species using alternative reagent ions (in this case NO^+^) as well as the more usual H_3_O^+^. This instrument is similar to the commercial SRI-ToF-MS instruments produced by Ionicon Analytik GmbH [42–43]. The theory behind PTR-MS has previously been described in detail [44–46]. The University of Innsbruck SRI-ToF-MS differs from the commercial instruments in that it uses a much higher sampling flow (ca. 500–1000 ml min^-1^ compared to 10–20 ml min^-1^), increasing the instrument’s ability to detect semi and low volatility compounds, and has a modified ion source which enables rapid switching between the different reagent ions.

While analyte ionisation by H_3_O^+^ takes place predominantly via proton transfer, chemical ionisation with NO^+^ can proceed via a number of chemical pathways. This enables SRI-ToF-MS to provide more structural information than traditional PTR-ToF-MS. For example an aldehyde and a ketone with the same sum formula (M) would be indistinguishable using PTR-ToF-MS as both would be detected at their protonated mass, MH^+^. However when ionised using NO^+^ the aldehyde will typically undergo hydride ion abstraction to give [M-H]^+^ and the ketone will cluster with NO^+^ to give MNO^+^, thereby allowing aldehydes and ketones to be measured individually [47].

The SRI-ToF-MS was operated in H_3_O^+^ mode with a drift tube pressure of 2.3 mbar, a drift tube and inlet line temperature of 60°C and with an applied voltage of 540 V, giving an E/N (the ratio of electric field strength (E) and the buffer gas number density (N)) of 120 Td (1 Td = 10^−21^ V m^-2^). When NO^+^ was used as the reagent ion the drift tube temperature and pressure remained the same but the applied voltage was reduced to 350 V, giving an E/N of 78 Td. E/N was reduced in the NO^+^ mode in order to facilitate cluster formation and therefore provide greater information with respect to the chemical structure of the analyte. The inlet line was heated to 70°C to prevent losses of semi-volatile compounds to internal surfaces.

The SRI-ToF-MS was operated with a measurement frequency of 1Hz and was switched from H_3_O^+^ to NO^+^ mode and vice-versa every 6 minutes. During the first measurement period, July-September 2013, the SRI-ToF-MS sampled for 24 minutes from the soil chamber, followed by 36 minutes from the leaf chamber before switching back to the soil chamber. As is discussed above, from April-June 2014 measurements were made from the leaf chamber alone.

### SRI-ToF-MS calibration and data analysis

Six point calibrations were carried out weekly using dynamic dilution of a gas standard (Apel-Riemer Environmental Inc.) comprising 20 BVOC species with protonated masses ranging from *m/z* 31 to 205. Mixing ratios within the gas standard were 1000 ppbv for all compounds with the exception of formaldehyde (5280 ppbv), acetonitrile (1186 ppbv), decanal (770 ppbv) and 1,3,5-triisopropylbenzene (666 ppbv) with a ± 5% uncertainty. Monoterpenes were calibrated using α-pinene and sesquiterpenes using 1,3,5-triisopropylbenzene. Compounds not present in the calibration gas standard were quantified using the sensitivity calculated for the closest calibrated mass with an equivalent dipole moment and oxidation state. Calibrations were carried out in H_3_O^+^ and NO^+^ modes and using both ambient and humidified air, the latter generated by passing ambient air through a water bubbler before catalytic purification. Humidification of the air, however, had little effect on the sensitivities calculated (except for formaldehyde). Mass scale calibration was enabled by the addition of either 1-chloro-2-iodobenzene or, in 2014, 1,2,4-trichlorobenzene to the sample air prior to the drift tube. SRI-ToF-MS data were analysed using the PTR-TOF Data Analyser described by Müller et al. [48] and references therein.

### GC-MS measurements

Sample collection for GC-MS analysis was performed throughout the 2014 measurement period to provide identification of isomeric terpene species. Samples for GC-MS analysis were taken from the leaf chamber outflow while the leaf chamber was exposed to clean (ozone free) air. Substantial losses of sesquiterpenes occur during sampling in the presence of ozone [49] as well as several artefacts [50], therefore GC-MS data are not available for the ozone fumigation period. GC-MS analysis was performed following established procedures [17, 51–53]. Air samples (30 L) were trapped at a flow rate of 200 ml min^-1^ onto glass tubes containing polydimethylsiloxane-foam-adsorbent material (Gerstel GmbH, Mülheim an der Ruhr, Germany). In previous studies the collection efficiencies of the sample tubes was found to be >99.7% for C5 compounds and >99% for C10 compounds when sampling 30L of BVOC at a mixing ratio of 1–10 ppb. Samples were then analysed using a thermo-desorption unit (TDU, Gerstel GmbH) coupled to a GC-MS (GC type: 7890A; S type: 5975C Agilent Technologies, Palo Alto, USA). BVOCs were separated using a 5% phenyl, 95% methylpolysiloxane capillary column (60 m × 250 μm × 0.25 μm DB-5MS + 10 m DG, Agilent Technologies) with a helium flow rate of 1.2 mL min^-1^ and a temperature programme of 40°C for 2 min, followed by ramping at 6°C min^-1^ to 80°C, holding for 3 min, ramping at 3.4°C min^-1^ to 170°C and finally at 12°C min^-1^ to 300°C before holding for 4 min. Calibration was achieved by injecting pure standard mixtures in hexane at seven different concentrations (20–800 pmol μl^−1^). Each concentration mixture was made independently in triplicate, and measured twice. The resulting MS signal responses were found to be linear with increasing standard concentrations (*r*^*2*^ = 0.978–0.999). Limits of detection (LOD) were set to twice σ, and the limit of quantification (LOQ) to 5-fold the LOD. Sensitivity changes during sample analysis were accounted for by the use of a fixed amount of δ-2-carene used as internal standard.

Compounds were identified by comparing the mass spectra obtained from samples and commercially available authentic standards (Sigma-Aldrich, Taufkirchen, Germany). When standards were not available, sample spectra were compared to those found in the 2011 National Institute of Standards and Technology Mass Spectral Library (NIST11) and Wiley library (v.275), and by comparing the non-isothermal Kovats retention indices (RI) calculated following standard procedure [54].

### Emission rate calculation

Volume mixing ratios (VMR, ppbv) were calculated from the raw SRI-ToF-MS data in H_3_O^+^ mode using the equation below.

$${VMR}_{BVOC}=\frac{I{\left(RH^{+}\right)}_{norm}}{\varepsilon_{norm}}$$ (1)

where I(RH^+^)_norm_ represents the count rate observed for each of the protonated BVOC species (I(RH^+^)), normalised to a primary ion (H_3_O^+^) and primary ion-water cluster (H_3_O^+^_._H_2_O) count of 10^6^ and background corrected. ε_norm_ is the normalised sensitivity at that mass, calculated by dynamic dilution of the gas standard. This method is based on that described by Tani et al. [55] and Taipale et al. [56] as applied by Acton et al. [57]. Concentrations (μg m^-3^) were then calculated from the volume mixing ratios using the ideal gas law.

The emission rate from the leaf chamber (nmol m^-2^ s^-1^) was calculated from the concentration, the constant flow rate into the chamber which was recorded daily and the leaf area which was calculated following the chamber experiments. Leaves were removed from plants and scanned, areas were then calculated using a custom program (freely available from https://sites.google.com/site/ptrtof/file-cabinet).

Statistical analysis was carried out with R [58]. The impact of ozone on BVOC emission from *B*. *napus* leaves was tested against the null hypothesis using a paired t-test. The test compared the mean emission of each compound in the 2 h prior to ozone fumigation against the mean emission during 2 h following ozone fumigation. The P-value has not been corrected for multiple testing.

## Results and discussion

### Below ground measurements

The principal BVOCs emitted from below ground were the sulfur compounds, CH_4_S, C_2_H_6_S_2_ and C_2_H_6_S, which are most probably methanethiol, dimethyl disulfide (DMDS) and dimethyl sulfide (DMS), respectively, in agreement with the observations made by van Dam et al. [59] using *Brassica* species. While the isothiocyanate marker reported by van Dam et al. [59] at m/z 60 was not observed, a mass spectral peak at m/z 74.0052 in the SRI-ToF-MS’s H_3_O^+^ mode was assigned tentatively to methyl thiocyanate.

Both monoterpene and sesquiterpene emissions have previously been reported from roots [60], but in the study presented here, significant emissions could only be detected following damage to the plant; these data are not shown here. The relatively high soil humidity during these experiments (46%) may, however, have impeded the detection of emitted mono- and sesqui-terpenes by hindering the transport of these hydrophobic species through the soil structure. Following ozone fumigation to the vegetation in the leaf chamber, we did not observe any significant changes to the BVOC emissions from below ground.

The BVOC emissions reported here may derive from both the roots of the plant and the associated micro-organisms in the rhizosphere. Below ground emissions from the soil and soil micro-organisms not associated with the plant are removed in the background correction. Micro-organisms present in soil and comparable habitats have been shown to be a source of numerous BVOC species including sulfur compounds [61–62] with the highest BVOC emission occurring as a result of anaerobic fermentation and metabolic processes [61]. A summary of the masses detected by the SRI-ToF-MS in H_3_O^+^ mode when sampling from the root chamber and their likely identities are shown in Table 2. The corresponding mass spectral peaks, where observed, in NO^+^ mode are also reported. Methanethiol, which has been shown to react slowly with NO^+^ [63] was not detected in NO^+^ mode. Mass spectral peaks in NO^+^ mode were assigned to compounds identified in H_3_O^+^ mode on the basis of their exact mass and agreement between the time traces recorded in each mode. As VOC-free air is introduced at the base of the chamber and passed up through the soil, thereby artificially increasing diffusion from the soil, the emission rates reported in Table 2 may be artificially elevated.

**Table 2:** BVOC species detected from below ground measurements following background correction against a soil filled chamber. Emission is compared with previous studies made using *Brassica* species.

m/z detected in H_3_O^+^ ionisation mode	m/z detected in NO^+^ ionisation mode	Molecular formula	Proposed compound	Emission—this study (nmol m^-2^ soil surface min^-1^)	Previously reported emission
49.011	not detected	CH_4_S	methanethiol	2.4	van Dam et al. [59]; Danner et al. [83]
63.026	62.017	C_2_H_6_S	dimethyl sulfide	5.8 × 10^−1^	van Dam et al.[59]; Danner et al. [83]
74.005	not detected	C_2_H_3_NS	methyl thiocyanate	1.9 × 10^−1^	
80.962	not detected	CH_4_S_2_	methanedithiol	9.0 × 10^−4^	
94.991	93.990	C_2_H_6_S_2_	dimethyl disulfide	6.4 × 10^−1^	van Dam et al.[59]; Danner et al. [83]
135.071	117.032	C_9_H_10_O	Unknown alcohol	9.4 × 10^−2^	

### Constitutive above-ground BVOC emission

**Fig 2:**
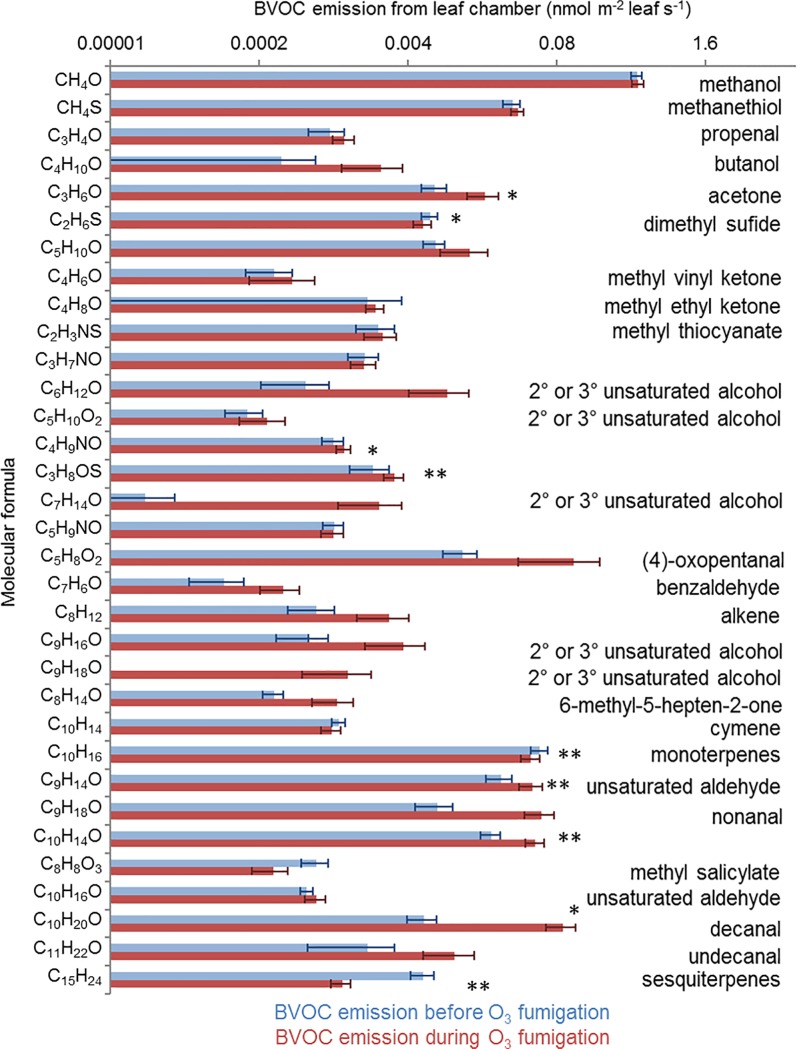
The principle leaf emitted BVOCs from *Brassica napus*. Blue bars represent BVOC emission prior to ozone fumigation and red bars represent BVOC emission during the first 2 h of ozone fumigation detected using SRI-ToF-MS and plotted on a log scale. BVOC species quantified from measurements carried out in H_3_O^+^ mode. Error bars represent the standard error in emission across 10 plants. * P < 0.05, ** P < 0.01 derived from a paired t-test comparing BVOC emission prior to ozone fumigation against BVOC emission during the first 2 h of ozone fumigation.

BVOC emissions from leaves of *B*. *napus* prior to ozone fumigation are summarised in Fig 2. BVOCs were quantified from measurements taken using the SRI-ToF-MS operated in H_3_O^+^ mode. NO^+^ ionisation was then used to provide additional structural information aiding the identification of BVOC species. Of the compounds emitted from above-ground leaf tissue: 25 contained oxygen, 4 contained nitrogen, 4 contained sulfur and 4 were pure hydrocarbons. The BVOC emissions from leaves prior to ozone fumigation were dominated by methanol and monoterpenes, with emission rates of 4.05 × 10^−1^ ± 4.33 × 10^−2^ (standard error) and 5.73 × 10^−2^ ± 9. 71 × 10^−3^ (standard error) nmol m^-2^ s^-1^ respectively. These compounds were detected at m/z 33.034 and 137.134 in the H_3_O^+^ spectrum, and at m/z 62.018 and 136.130 in the NO^+^ spectrum, suggesting that ionisation of methanol proceeded via cluster formation [64], and the ionisation of monoterpenes via charge transfer, as would be expected [65–66].

Large mass spectral peaks were also observed at *m/z* 49.011, 101.061, 139.114, 151.112, 153.053, 153.132 and 205.198 in the H_3_O^+^ spectrum and were tentatively assigned to methanethiol (CH_4_S), oxopentanal (C_5_H_8_O_2_) and an unknown aldehyde with the molecular formula C_9_H_14_O (as in both cases hydride ion abstraction is seen in the NO^+^ spectrum), an unsaturated oxygenated species with the molecular formula C_10_H_14_O, methyl salicylate (C_8_H_8_O_3_), an unsaturated aldehyde with the molecular formula C_10_H_14_O (as hydride ion abstraction is seen in the NO^+^ spectrum) and sesquiterpenes (C_15_H_24_) respectively. The emission of methyl salicylate from plants is usually associated with biotic [2, 67] or abiotic stress such as ozone exposure (5h, 120–170 ppb ozone, [14]), but low emissions (< 0.2 pmol m^-2^ s^-1^) have previously been reported from healthy tobacco plants as well [14].

GC-MS analysis of trapped BVOC samples was used to identify the species contributing to the monoterpenes and sesquiterpene peaks identified using the SRI-ToF-MS. The monoterpene emission was predominantly made up of limonene (90%, α-thujene (4%), α-pinene (4%), β-pinene (1%) and γ-terpinene (1%). The C_15_H_25_ signal was made up of four sesquiterpenes: β-elemene (43%), isolongifolene (15%), β-caryophyllene (36%) and α-farnesene (5%) together with the aromatic compound 1,3,5-tris(1-methylethyl) benzene (1%). The sesquiterpene emission rate calculated using the GC-MS (4.7 × 10^−2^ nmol m^-2^ s^-1^) agreed well with that calculated using the SRI-ToF-MS (5.5× 10^−2^ nmol m^-2^ s^-1^), but the total monoterpene emission rate calculated using the GC-MS was significantly lower than that recorded using the SRI-ToF-MS (6.2 × 10^−4^ and 5.7 × 10^−2^ nmol m^-2^ s^-1^ respectively). High levels of monoterpenes in GC-MS background samples suggest that an overestimation of the GC-MS background may have resulted in an underestimation of total monoterpene emission rate calculated using GC-MS. It is possible that the SRI-ToF-MS overestimated monoterpene emission rates due to the fragmentation of high mass compounds to m/z 137.134 when operated in H_3_O^+^ mode (for example, sesquiterpenes are known to fragment to this mass) [68]. However, as the monoterpene concentrations were consistent when the SRI-ToF-MS was operated in H_3_O^+^ and NO^+^ modes it is unlikely that fragmentation of high mass compounds to *m/z* 137 within the SRI-ToF-MS was responsible for an over estimation of monoterpene emission in this case.

Previous analysis of BVOC emissions from oilseed rape [35, 69–70] also reported significant emissions of monoterpenes and sesquiterpenes; however, the blend of individual monoterpenes and sesquiterpenes differs between these studies and ours. This is likely caused by changes in cultivar and growth stage. Ibrahim et al. [69] and McEwan and Macfarlane Smith [70] also reported a large emission of hexenyl-acetate, which was not detected in this study. This compound is associated with leaf wounding [21, 71] so emission may have been caused by leaf damage as result of leaf bagging prior to sampling in both studies.

### Inducted above-ground BVOC emission following ozone fumigation

Following fumigation of the *B*. *napus* leaves with ozone (ca. 140 ppbv) a burst of ca. 20 compounds was observed using SRI-ToF-MS operating in H_3_O^+^ mode. The emission rates of these compounds averaged over the first 2 h following ozone fumigation are displayed in Fig 2. Plant leaves were inspected following ozone fumigation experiments and no visible signs of damage were observed. In experiments where the analysis was repeated using an empty chamber, ozone loss to the chamber surface was found to be 42 ± 15 (standard error) ppbv or 16% of the inflowing ozone mixing ratio. When corrected for this loss to the chamber surface the average ozone loss to the plant was found to be 3.4 ± 0.3 (standard error) nmol per m^2^ leaf area s^-1^, giving a deposition velocity of 0.11 ± 0.01 (standard error) cm s^-1^. Carbon dioxide uptake and water loss from the plants remained stable during ozone fumigation, with average values of 7.4 ± 0.4 (standard error) μmol CO_2_ m^-2^ s^-1^ and 1.0 ± 0.1 (standard error) mmol H_2_O m^-2^ s^-1^ across all plants.

Previous investigations on the effect of ozone on plants have shown that ozone can cause a change in constitutive BVOC emissions from plants. Ryan et al. [15] observed a decrease in isoprene emissions within 2 days of ozone fumigation (120 ppbv) of an ozone-sensitive poplar genotype, but observed no change in the emission of isoprene following fumigation of an ozone-tolerant genotype. Heiden et al. [14], Vuorinen et al. [72] and Ghirardo et al. [20] reported an increase in terpene emissions following ozone fumigation (5h, 120–170 ppb; 4h, 150 ppb then 4h, 200 ppb; 1-2h, 800–900 ppb respectively) of pine, tobacco, lima bean, cotton, tomato and poplar, whereas Himanen et al. [35] observed a decrease in monoterpene emissions following ozone fumigation (8h a day for 16–19 days, 100 ppb) of oilseed rape. In this present study, no significant increase in monoterpene or sesquiterpene emission was observed directly after ozone fumigation ceased or during the following 24 hours. As was observed by Wildt et al. [73] however, ozone fumigation caused a significant increase in the emission of the saturated aldehyde decanal and an increase in nonanal.

During ozone fumigation, monoterpene and sesquiterpene emissions from the leaf chamber were observed to decrease immediately, by 17% and 82% respectively. Both monoterpenes and sesquiterpenes contain alkene functional groups and so would be expected to undergo ozonolysis via the mechanism proposed by Criegee [32]. Rate constants (k) for the gas phase ozonolysis (at room temperature) of the detected terpenes, where known, are shown in Table 3, together with expected half-lives (τ) at an ozone mixing ratio of 135 ppbv. Gas phase rate constants for the reaction of ozone with α-thujene and β-elemene have yet to be reported so these rate constants were estimated based on compounds with the same number of double bonds. The rate constant of β-elemene ozonolysis was taken to be 3.15 × 10^−15^ cm^3^ molecule^-1^ s^-1^ (the value calculated for β-ocimene by Kim et al. [74] and the rate constant for α-thujene was taken to be 8.4 × 10^−17^ cm^3^ molecule^-1^ s^-1^ (the value calculated for α -pinene by Lee et al. [75]). The calculated half-lives for ozonolysis range from 8.4 min to 5.6 h (Table 3). However, given the short residence time of air in the leaf chamber (ca. 6.5 min), a significant drop in monoterpene emissions as a result of gas phase ozonolysis can be discounted. While the gas phase ozonolysis of the more reactive sesquiterpenes, such as β-caryophyllene and α-farnesene, is likely to account for some of the loss observed, this is insufficient to explain the 82% decrease observed upon ozone fumigation.

**Table 3:** Gas phase rate coefficients (k) for the reaction of selected terpenes with ozone (2.46 molecules cm^-3^ s^-1^, 135 ppbv) at 296 K and their expected half-lives (τ).

Terpene	k_O3_	τ_O3_ (h)	Reference
limonene	2.0 × 10^−16^	0.4	Atkinson et al. [82]
α-pinene	8.4 × 10^−17^	1.0	Lee et al. [75]
β-pinene	1.5 × 10^−17^	5.6	Lee et al. [75]
γ-terpinene	1.4 × 10^−16^	0.6	Atkinson et al. [82]
β-caryophyllene	1.2 × 10^−16^	0.7	Shu and Atkinson [85]
isolongifolene	2.5 × 10^−17^	3.3	Ghalaieny et al. [84]
α-farnesene	5.9 × 10^−16^	0.1	Kim et al. [74]

The addition of an excess of an OH scavenger (cyclohexane) to the leaf chamber caused the monoterpene concentrations measured during ozone fumigation to return to the levels observed prior to ozone fumigation. This indicates that the drop in measured monoterpene emissions from the leaf chamber following ozone fumigation is a result of a reaction with OH radicals rather than direct reaction with ozone. A burst of cyclohexanone was observed during the period of cyclohexane addition indicating that OH was successfully scavenged. The addition of cyclohexane had no effect on the sesquiterpene emissions from the plant at the leaf chamber outlet, thereby demonstrating that the drop in sesquiterpene emissions from the leaf chamber is driven by ozonolysis. As has been shown by Fruekilde et al. [38] and Jud et al. [37], large, semi-volatile terpenoids may undergo ozonolysis on the leaf surface. The ozonolysis of sesquiterpenes, either before release from the plant surface, or after partition back into the condensed phase, could therefore account for a large proportion of the drop in sesquiterpene emissions from the leaf chamber not explained by gas phase ozonolysis.

**Fig 3:**
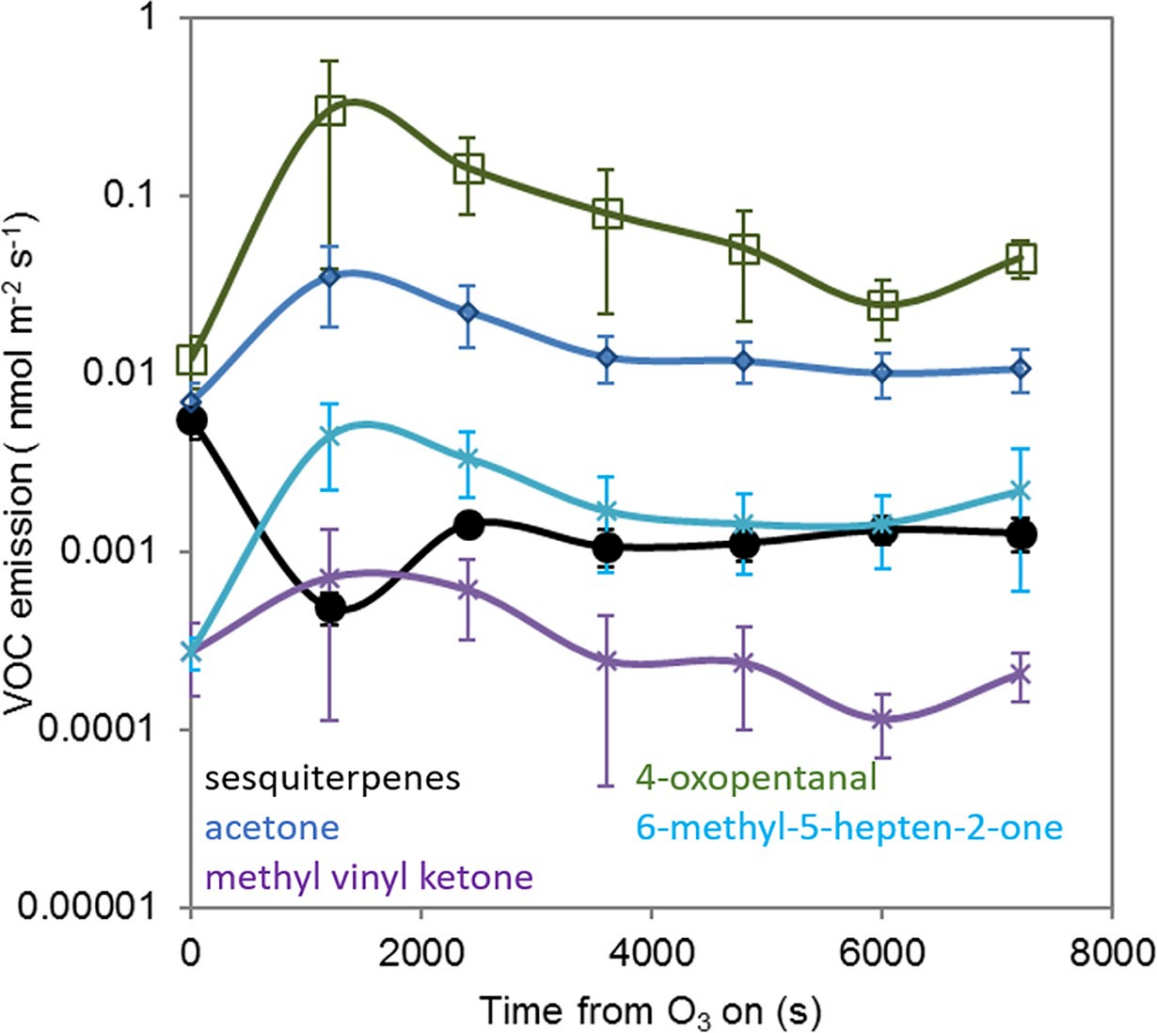
Sesquiterpene ozonolysis product formation following ozone fumigation. Black circles represent sesquiterpene emission from the leaf chamber (protonated *m/z* 205.198) following ozone exposure. Emission of the ozonolysis products acetone (protonated *m/z* 59.050), methyl vinyl ketone (protonated *m/z*, 71.050), oxopentanal (protonated *m/z* 101.061) and 6-methyl-5-hepten-2-one (protonated *m/z* 127.111), from the leaf chamber is represented by blue diamonds, purples crosses, green squares and turquoise crosses respectively. Error bars represent the standard error across 10 plants.

The reaction of ozone with molecules containing the alkene functional group to form a primary ozonide and the subsequent decomposition of this intermediate to a carbonyl and an energy rich carbonyl oxide intermediate was described by Criegee [32]. The so-called Criegee Intermediate (CI) may undergo a number of reactions including collisional stabilisation to form a stabilised CI. In the case of dialkyl-substituted or monosubstituted *syn*- CIs isomerisation leads to a “hot” hydroperoxide followed by decomposition to alkyl and OH radicals and in the case of unsubstituted or monosubstituted *anti*-CIs rearrangement leads to a “hot” ester followed by decomposition to CO_2_ and RH [33, 76]. The CIs and radical decomposition products are however expected to be short lived, so in order to observe sesquiterpene ozonolysis products we focused on the stable carbonyl products. The reactions of the cyclic sesquiterpenes β-caryophyllene [40], β-elemene and isolongifolene with ozone, yield formaldehyde and a range of large (> 204 amu) oxygenated compounds. Emissions of formaldehyde and these high molecular weight semi-volatile species were not observed during this study. The proton affinity of formaldehyde is only slightly higher than that of water, therefore at high humidity, the instrumental sensitivity of the SRI-ToF-MS operated in H_3_O^+^ reagent ion mode to formaldehyde is very low [77]. As the humidity in the leaf chamber was high it is therefore likely that if formaldehyde was produced it would not have been detected. It is likely that the large oxygenated products formed remain on the plant surface or are lost by deposition to the chamber walls and other surfaces [40]. These compounds are therefore not detected by SRI-ToF-MS in our experimental set-up. However, ozonolysis of the acyclic sesquiterpene α-farnesene leads to the formation of a number of short chain carbonyl compounds including acetone, methyl vinyl ketone, oxopentanal and 6-methyl-5-hepten-2-one, all of which were observed following ozone fumigation, supporting the suggestion that sesquiterpenes are lost by reaction with ozone (Fig 3). However, of these compounds only acetone showed a statistically significant change following ozone fumigation (P = 0.04). As these compounds contain an alkene functional group it is likely that they undergo further oxidation reducing the emission observed. Emissions of oxygenated BVOCs from plant surfaces during ozone exposure have also been observed by Karl et al. [78] who demonstrated that a number of compounds thought to be formed by gas phase chemistry within the canopy may also originate by reaction with ozone on leaf surfaces, and/or inside the leaf [79–80].

During ozone fumigation, total carbon emissions measured using SRI-ToF-MS (calculated by summing the moles of carbon emitted) from the leaf chamber increased from 1.65 nmol C m^-2^ leaf area s^-1^ to 3.93 nmol C m^-2^ leaf area s^-1^. The four α-farnesene ozonolysis products described above made up 23% of the increase in carbon emitted in the form of BVOCs from the leaf chamber. This large increase in carbon emitted in the form of BVOCs demonstrates that the increase in BVOCs observed following ozone exposure is a result of increased emission from the plant rather than simply the ozonolysis of the same BVOCs emitted by the plant prior to ozone exposure. These compounds are likely to be a result of liberation of carbon through ozonolysis of leaf surface compounds and/or the induced emission of oxygenated species from the plant itself. The leaf surface waxes of *B*. *napus* are predominantly made up of alkanes, saturated ketones, esters and alcohols [81] which are all relatively unreactive with ozone. Therefore, loss of ozone to the plant surface is likely to be via reaction with large, low volatility alkenes such as sesquiterpenes and homoterpenes which are known to be emitted by *B*. *napus* [35].

## Conclusions

Ozone fumigation of *B*. *napus* lead to an increase in BVOC emission from plant leaves of 2.3 nmol C m^-2^ leaf area s^-1^. This is approximately double the amount of carbon emitted in the form of BVOCs from unstressed plants. This increase in carbon emission from the leaf was predominantly in the form of aldehydes (60%), compounds containing aldehyde and ketone functional groups (22%), alcohols (5%) and ketones (2%).

The concentrations of sesquiterpenes in the air surrounding the plant was suppressed in the presence of ozone, but more so than can be explained by gas-phase ozonolysis alone. This suggests that 135 ppbv ozone alters the chemical signal emitted from the plant both by destroying sesquiterpenes in the gas phase but also by reactions occurring on the plant leaf surface. Hence, plant-plant and plant-insect signalling reliant on sesquiterpene emissions [23] may be disrupted in ozone-polluted air. It is not clear how our laboratory observations apply at the community or ecosystem scale, but we suggest that multi-trophic communication systems may be disturbed by elevated ozone concentrations in ambient air.

In contrast to the large change to above ground BVOC emissions following ozone fumigation, BVOC emission from plant roots was unaffected by ozone fumigation. BVOC signalling below ground [28] is therefore unlikely to be affected by elevated ozone concentrations in ambient air.

The data used in this study are available in the Lancaster University data repository (https://dx.doi.org/10.17635/lancaster/researchdata/218).

## References

[1] Dudareva N, Negre F, Nagegowda DA, Orlova I. Plant volatiles: Recent advances and future perspectives. Critical Reviews in Plant Sciences. 2006;25: 417–440.

[2] Holopainen JK, Gershenzon J. Multiple stress factors and the emission of plant VOCs. Trends in Plant Science. 2010;15(3): 176–184. 10.1016/j.tplants.2010.01.006 20144557 10.1016/j.tplants.2010.01.006

[3] Guenther AB, Jiang X, Heald CL, Sakulyanontvittaya T, Duhl T, Emmons LK, et al The Model of Emissions of Gases and Aerosols from Nature version 2.1 (MEGAN2.1): an extended and updated framework for modeling biogenic emissions. Geoscientific Model Development. 2012;5: 1471–1492.

[4] Atkinson R, Arey J. Gas-phase tropospheric chemistry of biogenic volatile organic compounds: a review. Atmospheric Environment. 2003;37(2): 197–219.

[5] Logan JA. Tropospheric ozone: Seasonal behavior, trends, and anthropogenic influence. Journal of Geophysical Research. 1985;90(D6): 10463–10482.

[6] Royal Society. Ground-level ozone in the 21st century: Future trends, impacts and policy implications: Science policy report 15/08. The Royal Society, London; 2008.

[7] Oltmans SJ, Lefohn AS, Shadwick D, Harris JM, Scheel HE, Galbally I, et al Recent tropospheric ozone changes–A pattern dominated by slow or no growth. Atmospheric Environment. 2013;67: 331–351.

[8] Fu Y, Tai APK. Impact of climate and land cover changes on tropospheric ozone air quality and public health in East Asia between 1980 and 2010. Atmospheric Chemistry and Physics. 2015;15: 10093–10106.

[9] Ashmore MR. Assessing the future global impacts of ozone on vegetation. Plant, Cell and Environment. 2005;28: 949–964.

[10] WHO. Health aspects of air pollution with particulate matter, ozone and nitrogen dioxide WHO, Copenhagen; 2003.

[11] Laothawornkitkul J, Taylor J, Paul ND, Hewitt CN. Biogenic volatile organic compounds in the Earth system. The New Phytologist. 2009;183: 27–51. 10.1111/j.1469-8137.2009.02859.x 19422541 10.1111/j.1469-8137.2009.02859.x

[12] Loreto F, Velikova V. Isoprene produced by leaves protects the photosynthetic apparatus against ozone damage, quenches ozone products, and reduces lipid peroxidation of cellular membranes. Plant Physiology. 2001;127: 1781–1787. 11743121 PMC133581

[13] Vickers CE, Possell M, Cojocariu CI, Velikova VB, Laothawornkitkul J, Ryan A, et al Isoprene synthesis protects transgenic tobacco plants from oxidative stress. Plant, Cell and Environment. 2008;35(5): 520–531. 10.1111/j.1365-3040.2009.01946.x 19183288

[14] Heiden AC, Hoffmann T, Kahl J, Kley D, Klockow D, Langebartels C, et al Emission of volatile organic compounds from ozone-exposed plants. Ecological Applications. 1999;9(4): 1160–1167.

[15] Ryan A, Cojocariu C, Possell M, Davies WJ, Hewitt CN. Defining hybrid poplar (*Populus deltoides* × *Populus trichocarpa*) tolerance to ozone: identifying key parameters. Plant, Cell and Environment 2009;32: 31–45. 10.1111/j.1365-3040.2008.01897.x 19076530 10.1111/j.1365-3040.2008.01897.x

[16] Fares S, Oksanen E, Lännenpää M, Julkunen-Tiitto R, Loreto F. Volatile emissions and phenolic compound concentrations along a vertical profile of Populus nigra leaves exposed to realistic ozone concentrations. Photosynthesis Research. 2010;104: 61–74. 10.1007/s11120-010-9549-5 20407831 10.1007/s11120-010-9549-5

[17] Behnke K, Kleist E, Uerlings R, Wildt J, Rennenberg H, Schnitzler J-P. RNAi-mediated suppression of isoprene biosynthesis in hybrid poplar impacts ozone tolerance. Tree Physiology. 2009;29: 725–736. 10.1093/treephys/tpp009 19324699 10.1093/treephys/tpp009

[18] Calfapietra C, Wiberley AE, Falbel TG, Linskey AR, Scarascia MG, Karnosky DF, et al Isoprene synthase expression and protein levels are reduced under elevated O3 but not under elevated CO2 (FACE) in field-grown aspen trees. Plant, Cell and Environment. 2007;30: 654–661. 10.1111/j.1365-3040.2007.01646.x 17407542 10.1111/j.1365-3040.2007.01646.x

[19] Fares S, Barta C, Brilli F, Centritto M, Ederli L, Ferranti F, et al Impact of high ozone on isoprene emission, photosynthesis and histology of developing Populus alba leaves directly or indirectly exposed to the pollutant. Physiologia Plantarum 2006;128: 456–465.

[20] Ghirardo A, Xie J, Zheng X, Wang Y, Grote R, Block K, et al Urban stress-induced biogenic VOC emissions and SOA-forming potentials in Beijing. Atmospheric Chemistry and Physics. 2016;16: 2901–2920.

[21] Maffei ME. Sites of synthesis, biochemistry and functional role of plant volatiles. South African Journal of Botany. 2010;76: 612–631.

[22] Dicke M. Behavioural and community ecology of plants that cry for help. Plant, Cell and Environment. 2009; 32: 654–665. 10.1111/j.1365-3040.2008.01913.x 19021885 10.1111/j.1365-3040.2008.01913.x

[23] Kessler A, Baldwin IT. Defensive function of herbivore-induced plant volatile emissions in nature. Science. 2001;291: 2141–2144. 10.1126/science.291.5511.2141 11251117 10.1126/science.291.5511.2141

[24] Gosset V, Harmel N, Göbel C, Francis F, Haubruge E, Wathelet J-P, et al Attacks by a piercing-sucking insect (*Myzus persicae Sultzer*) or a chewing insect (*Leptinotarsa decemlineata* Say) on potato plants (*Solanum tuberosum* L.) induce differential changes in volatile compound release and oxylipin synthesis. Journal of Experimental Botany. 2009;60(4): 1231–1240. 10.1093/jxb/erp015 19221142 10.1093/jxb/erp015 PMC2657539

[25] Laothawornkitkul J, Moore JP, Taylor JE, Possell M, Gibson TD, Hewitt CN, et al Discrimination of plant volatile signatures by an electronic nose: A potential technology for plant pest and disease monitoring. Environmental Science and Technology. 2008;42: 8433–8439. 19068829 10.1021/es801738s

[26] Degenhardt J, Hiltpold I, Köllner TG, Frey M, Gierl A, Gershenzon J, et al Restoring a maize root signal that attracts insect-killing nematodes to control a major pest. PNAS. 2009;106(32): 13213–13218. 10.1073/pnas.0906365106 19666594 10.1073/pnas.0906365106 PMC2726344

[27] Kappers IF, Aharoni A, van Herpen TWJM, Luckerhoff LLP, Dicke M, Bouwmeester HJ. Genetic engineering of terpenoid metabolism attracts bodyguards to Arabidopsis. Science. 2005;309: 2070–2072. 10.1126/science.1116232 16179482 10.1126/science.1116232

[28] Hiltpold I, Erb M, Robert CAM, Turlings TCJ. Systemic root signalling in a belowground, volatile-mediated tritrophic interaction. Plant, Cell and Environment. 2011;34: 1267–1275. 10.1111/j.1365-3040.2011.02327.x 21477121 10.1111/j.1365-3040.2011.02327.x

[29] Farré-Armengol G, Peñuelas J, Li T, Yli-Pirilä P, Filella I, Llusia J, et al Ozone degrades floral scent and reduces pollinator attraction to flowers. New Phytologist. 2015; 10.1111/nph.13620 26346807 10.1111/nph.13620

[30] Pinto DM, Blande JD, Souza SR, Nerg A-M, Holopainen JK. Plant volatile organic compounds (VOCs) in ozone (O_3_) polluted atmospheres: the ecological effects. Journal of Chemical Ecology. 2010;36: 22–34. 10.1007/s10886-009-9732-3 20084432 10.1007/s10886-009-9732-3

[31] Hewitt CN, Terry G. Understanding ozone plant chemistry. Environmental Science and Technology. 1992;26(10): 1890–1891.

[32] Criegee R. Mechanism of ozonolysis. Angewandte Chemie International Edition in English. 1975;14(11): 745–752.

[33] Atkinson R, Arey J. Atmospheric degradation of volatile organic compounds. Chemical Reviews. 2003;103: 4605–4638. 10.1021/cr0206420 14664626 10.1021/cr0206420

[34] Fischer T, Byerlee D, Edmeades G. Crop yields and global food security Australian Centre for International Agricultural Research, Canberra; 2014.

[35] Himanen SJ, Nerg A-M, Nissinen A, Pinto DM, Stewart CN, Poppy GM, et al Effects of elevated carbon dioxide and ozone on volatile terpenoid emissions and multitrophic communication of transgenic insecticidal oilseed rape (Brassica napus). The New Phytologist. 2009;181: 174–786. 10.1111/j.1469-8137.2008.02646.x 19076723 10.1111/j.1469-8137.2008.02646.x

[36] HGCA. HGCA descriptive list spring oilseed rape trials protocol 2012. HGCA, Kenilworth, Warwickshire; 2012 https://cereals.ahdb.org.uk/media/5275/protocol_-_sr2012_-_hgca_spring_oilseed_rape_trials_h2012.doc (last accessed 01.06.2018).

[37] Jud W, Fischer L, Canaval E, Wohlfahrt G, Tissier A, Hansel A. Plant surface reactions: an opportunistic ozone defence mechanism impacting atmospheric chemistry. Atmospheric Chemistry and Physics. 2016;16: 277–292.

[38] Fruekilde P, Hjorth J, Jensen NR, Kotzias D, Larsen B. Ozonolysis at vegetation surfaces: a source of acetone, 4-oxopentanal, 6-methyl-5-hepten-2-one, and geranyl acetone into the atmosphere. Atmospheric Environment. 1998;32(11): 1893–1902.

[39] Wisthaler A, Weschler CJ. Reactions of ozone with human skin lipids: sources of carbonyls, dicarbonyls, and hydroxycarbonyls in indoor air. Proceedings of the National Academy of Sciences. 2010;107(15): 6568–6575. 10.1073/pnas.0904498106 PMC2872416 19706436

[40] Winterhalter R, Herrmann F, Kanawati B, Nguyen TL, Peeters J, Vereecken L, et al The gas-phase ozonolysis of beta-caryophyllene (C(15)H(24)). Part I: an experimental study. Physical Chemistry Chemical Physics. 2009;11: 4152–4172. 10.1039/b817824k 19458818 10.1039/b817824k

[41] Graus M, Müller M, Hansel A. High resolution PTR-TOF: quantification and formula confirmation of VOC in real time. Journal of the American Society for Mass Spectrometry. 2010;21: 1037–1044. 10.1016/j.jasms.2010.02.006 20335047 10.1016/j.jasms.2010.02.006

[42] Jordan A, Haidacher S, Hanel G, Hartungen E, Herbig J, Märk L, et al An online ultra-high sensitivity Proton-transfer-reaction mass-spectrometer combined with switchable reagent ion capability (PTR+SRI−MS). International Journal of Mass Spectrometry. 2009;286(1): 32–38.

[43] Jordan A, Haidacher S, Hanel G, Hartungen E, Märk L, Seehauser H, et al A high resolution and high sensitivity proton-transfer-reaction time-of-flight mass spectrometer (PTR-TOF-MS). International Journal of Mass Spectrometry. 2009;286(2–3): 122–128.

[44] Hansel A, Jordan A, Holzinger R, Prazeller P, Vogel W, Lindinger W. Proton transfer reaction mass spectrometry: on-line trace gas analysis at the ppb level. International Journal of Mass Spectrometry and Ion Processes. 1995;149/150: 609–619.

[45] Hewitt CN, Hayward S, Tani A. The application of proton transfer reaction-mass spectrometry (PTR-MS) to the monitoring and analysis of volatile organic compounds in the atmosphere. Journal of Environmental Monitoring. 2003;5: 1–7. 12619749 10.1039/b204712h

[46] Lindinger W, Hansel A, Jordan A. On-line monitoring of volatile organic compounds at pptv levels by means of Proton-Transfer-Reaction Mass Spectrometry (PTR-MS) Medical applications, food control and environmental research. International Journal of Mass Spectrometry and Ion Processes. 1998;73: 191–241.

[47] Španěl P, Ji Y, Smith D. SIFT studies of the reactions of H_3_O^+^, NO^+^ and O2+; with a series of aldehydes and ketones. International Journal of Mass Spectrometry and Ion Processes. 1997;165/166: 25–37.

[48] Müller M, Mikoviny T, Jud W, D’Anna B, Wisthaler A. A new software tool for the analysis of high resolution PTR-TOF mass spectra. Chemometrics and Intelligent Laboratory Systems. 2013;127: 158–165.

[49] Pollmann J, Ortega J, Helmig D. Analysis of atmospheric sesquiterpenes: Sampling losses and mitigation of ozone interferences. Environmental Science and Technology. 2005;39: 9620–9629. 16475343 10.1021/es050440w

[50] Lee JH, Batterman SA, Jia C, Chernyak S. Ozone artefacts and carbonyl measurements using Tenax GR, Tenax TA, Carbopack B, and Carbopack X adsorbents. Journal of the Air & Waste Management Association. 2006;56: 1503–1517. 17117735 10.1080/10473289.2006.10464560

[51] Ghirardo A, Heller W, Fladung M, Schnitzler J-P, Schroeder H. Function of defensive volatiles in pedunculate oak (*Quercus robur*) is tricked by the moth *Tortrix viridana*. Plant, Cell and Environment. 2012;35(12): 2192–2207. 10.1111/j.1365-3040.2012.02545.x 22632165 10.1111/j.1365-3040.2012.02545.x

[52] Kreuzwieser J, Scheerer U, Kruse J, Burzlaff T, Honsel A, Alfarraj S, et al The Venus flytrap attracts insects by the release of volatile organic compounds. Journal of Experimental Botany. 2014;65: 755–766. 10.1093/jxb/ert455 24420576 10.1093/jxb/ert455 PMC3904726

[53] Weikl F, Ghirardo A, Schnitzler J-P, Pritsch K. Sesquiterpene emissions from *Alternaria alternata* and *Fusarium oxysporum*: Effects of age, nutrient availability, and co-cultivation. Scientific Reports. 2016;6: 22152 10.1038/srep22152 26915756 10.1038/srep22152 PMC4768142

[54] Gonzalez FR, Nardillo AM. Retention index in temperature-programmed gas chromatography. Journal of Chromatography A. 1999;842: 29–49.

[55] Tani A, Hayward S, Hansel A, Hewitt CN. Effect of water vapour pressure on monoterpene measurements using proton transfer reaction-mass spectrometry (PTR-MS). International Journal of Mass Spectrometry. 2004;239: 161–169.

[56] Taipale R, Ruuskanen TM, Rinne J, Kajos MK, Hakola H, Pohja T, et al Technical Note: Quantitative long-term measurements of VOC concentrations by PTR-MS measurement, calibration, and volume mixing ratio calculation methods. Atmospheric Chemistry Physics. 2008;8: 6681–6698.

[57] Acton WJF, Schallhart S, Langford B, Valach A, Rantala P, Fares S, et al Canopy-scale flux measurements and bottom-up emission estimates of volatile organic compounds from a mixed oak and hornbeam forest in northern Italy. Atmospheric Chemistry and Physics. 2016;16: 7149–7170.

[58] R Core Team. R: A language and environment for statistical computing R Foundation for Statistical Computing, Vienna, Austria 2012; ISBN 3-900051-07-0, URL http://www.R-project.org/.

[59] van Dam NM, Samudrala D, Harren FJM, Cristescu SM. Real-time analysis of sulfur-containing volatiles in Brassica plants infested with root-feeding Delia radicum larvae using proton-transfer reaction mass spectrometry. AoB Plants. 2012;pls021. 10.1093/aobpla/pls021 PMC3424660 22916330

[60] Lin C, Owen S, Penuelas J. Volatile organic compounds in the roots and rhizosphere of *Pinus spp*. Soil Biology and Biochemistry. 2007;39: 951–960.

[61] Insam H, Seewald MSA. Volatile organic compounds (VOCs) in soils. Biology and Fertility of Soils. 2010;46: 199–213.

[62] Mayrhofer S, Mikoviny T, Waldhuber S, Wagner AO, Innerebner G, Franke-Whittle IH, et al Microbial community related to volatile organic compound (VOC) emission in household biowaste. Environmental Microbiology. 2006;8(11): 1960–1974. 10.1111/j.1462-2920.2006.01076.x 17014495 10.1111/j.1462-2920.2006.01076.x

[63] Pysanenko A, Španěl P, Smith D. A study of sulfur-containing compounds in mouth- and nose-exhaled breath and in the oral cavity using selected ion flow tube mass spectrometry. Journal of Breath Research. 2008;046004:13pp. 10.1088/1752-7155/2/4/046004 21386191

[64] Španěl P, Smith D. SIFT studies of the reactions of H_3_O^+^, NO^+^ and O2+ with a series of alcohols. International Journal of Mass Spectrometry and Ion Processes. 1997;167/168: 375–388.

[65] Diskin AM, Wang T, Smith D, Španěl PA. selected ion flow tube (SIFT), study of the reactions of H_3_O^+^, NO^+^ and O2+ ions with a series of alkenes; in support of SIFT-MS. International Journal of Mass Spectrometry. 2002;218: 87–101.

[66] Wang T, Španěl P, Smith D. Selected ion flow tube, SIFT, studies of the reactions of H_3_O^+^, NO^+^ and O2+ with eleven C_10_H_16_ monoterpenes. International Journal of Mass Spectrometry. 2003;228: 117–126.

[67] Pinto DM, Blande JD, Nykänen R, Dong W-X, Nerg A-M, Holopainen JK. Ozone degrades common herbivore-induced plant volatiles: does this affect herbivore prey location by predators and parasitoids? Journal of Chemical Ecology. 2007;33(4): 683–694. 10.1007/s10886-007-9255-8 17333375 10.1007/s10886-007-9255-8

[68] Kim S, Karl T, Helmig D, Daly R, Rasmussen R, Guenther A. Measurement of atmospheric sesquiterpenes by proton transfer reaction-mass spectrometry (PTR-MS). Atmospheric Measurement Techniques. 2009;2: 99–112.

[69] Ibrahim MA, Stewart-Jones A, Pulkkinen J, Poppy GM, Holopainen JK. The influence of different nutrient levels on insect-induced plant volatiles in Bt and conventional oilseed rape plants. Plant Biology. 2008;10: 97–107. 10.1111/j.1438-8677.2007.00013.x 18211550 10.1111/j.1438-8677.2007.00013.x

[70] McEwan M, Macfarlane Smith WH. Identification of volatile organic compounds emitted in the field by oilseed rape (*Brassica napus* ssp. oleifera) over the growing season. Clinical and Experimental Allergy. 1998;28: 332–338. 9543083 10.1046/j.1365-2222.1998.00234.x

[71] Fall R, Karl T, Hansel A, Jordan A, Lindinger W. Volatile organic compounds emitted after leaf wounding: On-line analysis by proton-transfer-reaction mass spectrometry. Journal of Geophysical Research. 1999;104(D13): 15963–15974.

[72] Vuorinen T, Nerg A-M, Holopainen JK. Ozone exposure triggers the emission of herbivore-induced plant volatiles, but does not disturb tritrophic signalling. Environmental Pollution. 2004;131: 305–311. 10.1016/j.envpol.2004.02.027 15234097 10.1016/j.envpol.2004.02.027

[73] Wildt J, Kobel K, Schuh-Thomas G, Heiden AC. Emissions of oxygenated volatile organic compounds from plants part II: Emissions of saturated aldehydes. Journal of Atmospheric Chemistry. 2003;45: 173–196.

[74] Kim D, Stevens PS, Hites RA. Rate constants for the gas-phase reactions of OH and O_3_ with β-ocimene, β-myrcene and α- and β-farnesene as a function of temperature. Journal of Physical Chemistry A. 2011;115: 500–506. 10.1021/jp111173s 21166436

[75] Lee A, Goldstein AH, Keywood MD, Gao S, Varutbangkul V, Bahreini R, et al Gas-phase products and secondary aerosol yields from the ozonolysis of ten different terpenes. Journal of Geophysical Research. 2006;111(D7): D07302.

[76] Kroll JH, Sahay SR, Anderson JG, Demerjian KL, Donahue NM. Mechanism of HO_x_ formation in the gas-phase ozone-alkene Reaction. 2. Prompt versus thermal dissociation of carbonyl oxides to form OH. Journal of Physical Chemistry A. 2001;105: 4446–4457. 10.1021/jp002121r 34500545

[77] de Gouw J, Warneke C. Measurements of volatile organic compounds in the earth's atmosphere using proton transfer-reaction mass spectrometry. Mass Spectrometry Reviews. 2007;26: 223–257. 10.1002/mas.20119 17154155 10.1002/mas.20119

[78] Karl T, Harley P, Guenther A, Rasmussen R, Baker B, Jardine K, et al The bi-directional exchange of oxygenated VOCs between a loblolly pine (*Pinus taeda*) plantation and the atmosphere. Atmospheric Chemistry and Physics. 2005;5: 3015–3031.

[79] Niinemets Ü, Fares S, Harley P, Jardine KJ. Bidirectional exchange of biogenic volatiles with vegetation: Emission sources, reactions, breakdown and deposition. Plant, Cell and Environment. 2014;37: 1790–1809. 10.1111/pce.12322 24635661 10.1111/pce.12322 PMC4289707

[80] Salter L, Hewitt CN. Ozone-hydrocarbon interactions in plants. Phytochemistry. 1992;31(12): 4045–4050.

[81] Holloway PJ, Brown GA, Baker EA, Macey MJK. Chemical composition and ultrastructure of the epicuticular wax in three lines of Brassica napus (L). Chemistry and Physics of Lipids. 1977;19: 114–127.

[82] Atkinson R, Hasegawa D, Aschmann SM. Rate constants for the gas-phase reactions of O3 with a series of monoterpenes and related compounds at 296 ± 2 K. International Journal of Chemical Kinetics. 1990;22: 871–887.

[83] Danner H, Samudrala D, Cristescu SM, van Dam NM. Tracing Hidden Herbivores: Time-Resolved Non-Invasive Analysis of Belowground Volatiles by Proton-Transfer-Reaction Mass Spectrometry (PTR-MS). Journal of Chemical Ecology. 2012;38: 785–794. 10.1007/s10886-012-0129-3 22592334 10.1007/s10886-012-0129-3 PMC3375075

[84] Ghalaieny M, Bacak A, McGillen M, Martin D, Knights AV, O'Doherty S, et al Determination of gas-phase ozonolysis rate coefficients of a number of sesquiterpenes at elevated temperatures using the relative rate method. Physical Chemistry Chemical Physics. 2012;14: 6596–6602. 10.1039/c2cp23988d 22456861 10.1039/c2cp23988d

[85] Shu Y, Atkinson R. Rate constants for the gas-phase reactions of O_3_ with a series of terpenes and OH radical formation from the O_3_ reactions with sesquiterpenes at 296 ± 2 K. International Journal of Chemical Kinetics. 1994;26: 1193–1205.

